# Cytoplasmic Translocation of Nucleolar Protein NOP53 Promotes Viral Replication by Suppressing Host Defense

**DOI:** 10.3390/v10040208

**Published:** 2018-04-20

**Authors:** Wen Meng, Shi-Chong Han, Cui-Cui Li, Hui-Jun Dong, Jian-Yu Chang, Hwa-Chain Robert Wang, Xiao-Jia Wang

**Affiliations:** 1 Key Laboratory of Animal Epidemiology of the Ministry of Agriculture, College of Veterinary Medicine, China Agricultural University, Beijing 100193, China; mengwen422fei@163.com (W.M.); hanshichong081@126.com (S.-C.H.); piqiubang@163.com (C.-C.L.); donghuijun105@163.com (H.-J.D.); 2 Department of Basic Veterinary, College of Veterinary Medicine, China Agricultural University, Beijing 100193, China; changjianyu@cau.edu.cn; 3 Department of Biomedical and Diagnostic Sciences, College of Veterinary Medicine, The University of Tennessee, Knoxville, TN 37996, USA; hcrwang@utk.edu

**Keywords:** NOP53, cytoplasmic translocation, recombinant protein, viral replication, IFN-β

## Abstract

NOP53 is a tumor suppressor protein located in the nucleolus and is translocated to the cytoplasm during infection by vesicular stomatitis virus (VSV) and herpes simplex virus type 1 (HSV-1), as shown in our previous study. Cytoplasmic NOP53 interacts with the retinoic acid-inducible gene I (RIG-I) to remove its K63-linked ubiquitination, leading to attenuation of type I interferon IFN-β. In the present study, we found no obvious translocation of NOP53 in infection by a mutant virus lacking ICP4 (HSV-1/d120, replication inadequate). Blocking cytoplasmic translocation of NOP53 by the deletion of its nuclear export sequence (NES) abrogated its ability to support viral replication. These results demonstrated that NOP53 redistribution is related to viral replication. It is interesting that treatment with poly (I:C) or RIG-I-N (a constitutively-active variant) directly induced NOP53 cytoplasmic translocation. To better assess the function of cytoplasmic NOP53 in viral replication, the NOP53-derived protein N3-T, which contains a human immunodeficiency virus (HIV)-derived cell-penetrating Tat peptide at the C-terminal region of N3 (residues 330–432), was constructed and expressed. The recombinant N3-T protein formed trimers, attenuated the expression of IFN-β and IFN-stimulated genes, as well as decreased the phosphorylation level of interferon regulatory factor 3 (IRF3). Furthermore, N3-T promoted the efficient replication of enveloped and non-enveloped DNA and RNA viruses belonging to 5 families. Our findings expand the understanding of the mechanism by which viruses utilize the nucleolar protein NOP53 for optimal viral replication.

## 1. Introduction

Innate immunity is critical for defending the host from pathogens, and type I interferon (IFN) is the core of the cellular antiviral response [1,2,3,4,5]. Viral infections produce highly conserved pathogen-associated molecular patterns (PAMPs) such as double-stranded RNA (dsRNA). Viral RNA contains short regions of hairpin dsRNA with triphosphorylated 5′ ends (5′ppp-RNA) that preferentially activate host pattern recognition receptors (PRRs) such as retinoic acid inducible gene-I (RIG-I) and related receptors [6]. RIG-I, a member of the RIG-I-like receptor (RLR) family, interacts with viral RNA and recruits mitochondrial-associated virus stimulators (MAVS—also known as IPS-1/Cardif/VISA) [7,8]. MAVS recruits tumor necrosis receptor-associated factor 3 (TRAF3), which causes TRAF3 lysine 63 (K63)-linked auto-ubiquitination to provide docking sites for the TANK binding kinase 1/I kappa-B kinase epsilon (TBK1/IKKε) complex [9,10,11]. This complex undergoes auto-phosphorylation-mediated activation, resulting in phosphorylation and activation of the type I IFN regulatory factor IRF3 and/or IRF7 to induce IFN gene expression [4]. IFNs secreted from virus-infected cells bind to IFN receptors (IFNARs) on the cell surface to activate the JAK-STAT pathway, leading to the upregulation of hundreds of IFN-stimulated gene products (ISGs) to suppress viral infection [12,13]. One RIG-I agonist has been shown to induce IFN expression and multiple innate antiviral responses to control viral infections, for example, vesicular stomatitis virus (VSV) of the family *Rhabdoviridae* [14] and the herpes simplex virus 1 (HSV-1) of the family *Herpesviridae* [15].

The nucleolar protein NOP53 (also designated PICT-1/GLTSCR2) is a tumor suppressor. Decreased NOP53 is correlated with cancer malignancy [16,17,18,19]. Knockdown of NOP53 results in the degradation of PTEN, a major negative regulator of the PI3K-Akt pathway, to inhibit PTEN-modulated apoptosis [20,21]. Suppression of NOP53 also sensitizes cells to DNA damage, delays DNA repair and abolishes G2/M checkpoint activation [22]. Localization of NOP53 is mediated by multiple unique nucleolar localization sequences [23]. Our previous study showed that VSV infection induces NOP53 translocation from the nucleus. Cytoplasmic NOP53 negatively regulates RIG-I via K63-linked ubiquitination, leading to inactivation of RIG-I and blockage of IFN-β induction [24]. We also found that NOP53 migrates from the nucleus of HSV-1 infected cells and that ectopic expression of viral protein γ34.5 facilitates the redistribution of NOP53 in response to infection by γ34.5 deleted HSV-1 (Δγ34.5) [25].

In the present study, we explored the underlying stimulus or host modulators that are related to NOP53 cytoplasmic translocation. We also expressed and purified the NOP53-derived protein N3-T to further examine the function of cytoplasmic NOP53 in the replication of DNA and RNA viruses from distinct genera. Our findings advance current knowledge of negative modulator of type I IFN, especially those that translocate from the nucleus to the cytoplasm, to support viral replication.

## 2. Materials and Methods

### 2.1. Cells and Viruses

HeLa, Vero, RD, PK-15, and HEK293T cells were cultured in Dulbecco’s modified Eagle’s medium (DMEM) and supplemented with 2 mM l-glutamine, nonessential amino acids, pyruvate sodium, 10% heat-inactivated fetal bovine serum (FBS), 100 U/mL penicillin and 100 µg/mL streptomycin (all reagents were purchased from Gibco Invitrogen, Carlsbad, CA, USA). All cells were cultured at 37 °C in a humidified incubator with 5% CO_2_. Herpes simplex virus 1 (HSV-1) strain F, mutant viruses d120 [26] and Δγ34.5 [25], vesicular stomatitis virus (VSV) strain Indiana [24], canine distemper virus (CDV) strain MD77 [27], enterovirus A71 (EV-A71) strain BC08 [28], pseudorabies virus (PRV) strain Fa [29], foot-and-mouth disease virus (FMDV) strain O [30], and porcine epidemic diarrhea virus (PEDV) strain CV777 [31] were reproduced in Vero, HEK293T, Vero, RD, Vero, PK-15, and Vero cells, respectively, according to the reports indicated above.

### 2.2. Immunofluorescence Studies

Cells growing on glass coverslips were fixed with 4% paraformaldehyde for 15 min, quenched with 100 mM glycine for 15 min, permeabilized with 0.1% Triton X-100 for 15 min, and blocked with phosphate-buffered saline (PBS) containing 10% horse serum plus 1% bovine serum albumin (BSA) for 2 h. The cells were washed three times with PBS and incubated with diluted primary antibodies overnight at 4 °C. Cells were washed with PBS three times at room temperature, then incubated with fluorochrome-conjugated secondary antibodies in the dark for 1 h at room temperature. The cells were then rinsed and incubated with 4′,6-diamidino-2-phenylindole (DAPI; Beyotime Biotechnology, Haimen, China) for 5 min, rinsed, mounted and examined; images were captured using an Olympus FluoView^TM^ FV1000. The primary antibodies used were as follows: rabbit polyclonal antibody to NOP53 (1:1000, Abcam, Cambridge, MA, USA); mouse monoclonal antibodies to HSV-ICP8 (1:500, Santa Cruz lnc., Santa Cruz, CA, USA), GFP (1:1000, Beyotime Biotechnology), His (1:1000, Beyotime Biotechnology), CDV-N (1:1000, Santa Cruz lnc.). The secondary antibodies used were as follows: goat anti-rabbit IgG (H+L) secondary antibody conjugated to Alexa Flour 594 (Invitrogen, Carlsbad, CA, USA) and goat anti-mouse IgG (H+L) secondary antibody conjugated to Alexa Flour 488 (Invitrogen); the images were processed using FV10-ASW 4.0 software (Olympus, Tokyo, Japan).

### 2.3. Construction and Transfection of NOP53 and Its Mutant

The vectors pFlag-CMV3 and pEGFP-N1 were purchased from Clontech. NOP53 and NES mutant (ΔNOP) were cloned using specific primers. Eukaryotic expression plasmids containing the genes were transfected into cells with the aid of Lipofectamine^TM^ 2000 (Invitrogen) according to the manufacturer’s instructions.

### 2.4. Preparation of Cell Lysates and Immunoblots

Cells were harvested at the times indicated by scraping and were collected by centrifugation. The cells were then rinsed with PBS, dissolved in 200 µL RIPA lysis buffer containing a protease inhibitor cocktail (Roche, Basel, Switzerland) and disrupted by sonication. Proteins harvested from cells were solubilized, separated by 12% sodium dodecyl sulfate-polyacrylamide gel electrophoresis (SDS-PAGE) and transferred to polyvinylidene difluoride (PVDF) membranes (Millipore, Burlington, MA, USA). The membranes were pretreated with 5% milk and reacted with appropriate primary antibodies, including rabbit polyclonal antibodies to NOP53 (1:1000, Abcam), IRF3 (1:800, Cell Signaling Technology, Danvers, MA, USA), p-IRF3 (1:800, Cell Signaling Technology), PTEN (1:1000, Beyotime Biotechnology), LC3B (1:1000, Beyotime Biotechnology), PARP (1:1000, Beyotime Biotechnology), mouse monoclonal antibody to Flag (1:1000, Beyotime Biotechnology), Histone H3 (1:1000, Beyotime Biotechnology), actin (1:1000, Beyotime Biotechnology), GFP (1:1000, Beyotime Biotechnology), His (1:1000, Beyotime Biotechnology), ICP0 (1:500, Santa Cruz), ICP8 (1:500, Santa Cruz), VSV-G (1:1000, Santa Cruz), CDV-N (1:1000, Santa Cruz) Beclin-1 (1:1000, Santa Cruz) and PEDV-N (1:1000, Alpha Diagnostic, San Antonio, TX, USA). The protein bands were detected using secondary antibodies including goat anti-mouse secondary antibodies conjugated to horseradish peroxidase (HRP) (Beyotime Biotechnology), goat anti-rabbit secondary antibodies conjugated to HRP (Beyotime Biotechnology). Actin serves as a loading control. Histone H3 served as a loading control for separated nuclear proteins.

### 2.5. LMB Treatment

HeLa cells were transfected with NOP53 for 36 h and then exposed to HSV-1 at a multiplicity of infection (MOI) of 10 in the presence or absence of LMB at 12 h after infection. The cells were then fixed at 1 or 2 h after treatment followed by immunofluorescence studies.

### 2.6. Cytoplasmic and Nuclear Fractionation

Cells were transfected with control (Ctrl) plasmid, plasmids that expressed Flag-tagged wt NOP53 (NOP53) or NES deletion mutant (ΔNOP53). The cells were then mock-infected or exposed to HSV-1/F at an MOI of 10. Cytoplasmic and nuclear fractions were then isolated using a kit (Pierce, Rockford, IL, USA, No. 78833) and analyzed by immunoblotting with appropriate primary and secondary antibodies.

### 2.7. Determination of 50% Tissue Culture Infective Dose (TCID_50_)

Cells were exposed to VSV, PEDV, or PRV in DMEM. After 90 min, the inoculum was replaced with DMEM containing 1% FBS followed by 48 h or 6 days (only for PEDV) of incubation. Virus titration was performed in HEK293T (only for VSV) or Vero cells seeded on 96-well plates at 10^4^ cells/well. Tenfold serial dilution was prepared for each sample, and 100 µL/well of each dilution was added to the cells in quadruplicate. The cytopathic effect was scored as positive for virus growth and log_10_ TCID_50_/mL was determined using the Reed-Muench method.

### 2.8. Protein Expression and Purification

N3 and N3-T were constructed and cloned into the expression vector pET-28a using the NdeI-EcoRI restriction sites. *Escherichia coli* (*E. coli*) strain BL21 (DE3) transformed with the recombinant pET-28a-N3 or N3-T plasmid was grown at 37 °C in a Luria-Bertani liquid medium to an optical density of 0.8 (OD at 590 nm) before being induced with 1 mM isopropyl β-d-1-thiogalactopyranoside (IPTG) for 4 h. The cells were harvested and lysed by sonication in PBS (pH 7.3) containing 1 mM phenylmethanesulfonyl fluoride (PMSF), and the supernatants were passed over a His-Ni column. The N3 and N3-T His fusion proteins were eluted and concentrated by ultrafiltration using 5 K membranes (Millipore) and were stored at −80 °C.

### 2.9. Chemical Cross-Linking

Purified proteins were incubated at 4 °C in the presence of Glutaraldehyde (GA, MP Biomedicals, Santa Ana, CA, USA) for 15 min. The reaction was terminated by the addition of an SDS-loading buffer. Cross-linking products were analyzed with immunoblotting.

### 2.10. RNA Extraction and Quantitave Real-Time Polymerase Chain Reaction

Replicated cultures were harvested and total RNA was extracted with Trizol (Invitrogen). A two-step real-time polymerase chain reaction (RT-PCR) was performed using an SYBR green supermix (Toyobo, Osaka Prefecture, Japan) as previously described [24]. The primers used were as follows: *IFN-β*: 5′-CCTACAAAGAAGCAGCAA-3′ (forward), 5′-TCCTCAGGGATGTCAAAG-3′ (reverse). *PKR*: 5′-TACGCCTGACCACAACTA-3′ (forward), 5′-GGTATTCCTTCCCGTCTA-3′ (reverse). *ISG56*: 5′-CACCCACTTCTGTCTTACT-3′ (forward), 5′-ACATTCTTGCCAGGTCTA-3′ (reverse). The primers for *GAPDH* have been previously described [24].

### 2.11. Cytotoxicity Assay

Protein cytotoxicity was assessed by lactate dehydrogenase (LDH) assay with a Cytotox-One^TM^ homogenous membrane integrity kit (Promega, Madison, WI, USA), according to the manufacturer’s instructions.

### 2.12. Statistics

All data were determined in triplicate and expressed as the means and standard deviations (SD). Statistical analysis was performed using a two-way ANOVA with Prism 5.01 (GraphPad Software, La Jolla, CA, USA). Tukey’s multiple comparison tests were used to analyze the differences between means.

## 3. Results

### 3.1. Cytoplasmic Translocation of NOP53 Plays an Important Role in Support of HSV-1 Replication

**Figure 1:**
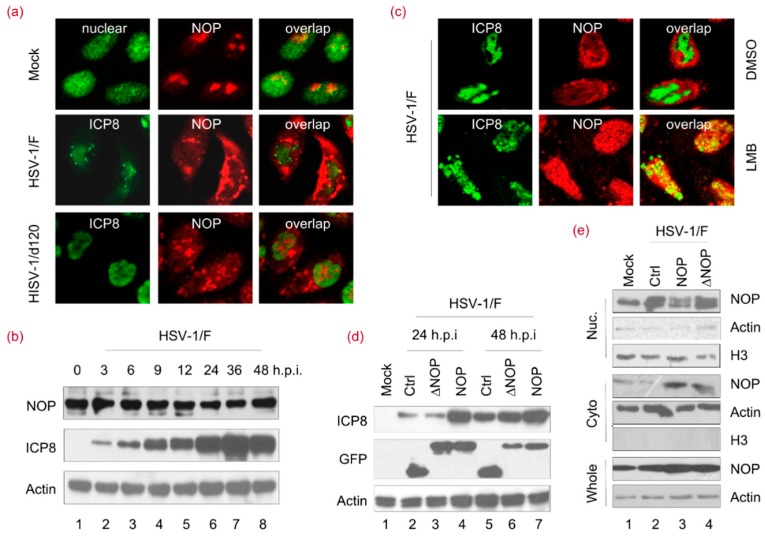
Cytoplasmic translocation of NOP53 plays an important role in the support of HSV-1 replication. (**a**) HeLa cells were mock-infected or exposed to 10 multiplicity of infection (MOI) of the herpes simplex virus (HSV-1/F) or d120 for 12 h. The cells were fixed and then stained with antibodies against ICP8 and NOP53 (NOP), and images were merged. All images were taken using 100× objectives with an Olympus FluoView^TM^. ICP8 localizes in the nucleus and served to demarcate this structure [26]; (**b**) HeLa cells were infected with HSV-1/F at 1 MOI for 0, 3, 6, 9, 12, 24, 36, or 48 h; cell lysates were prepared, and NOP53 and HSV-ICP8 were measured by immunoblotting with specific antibodies, using actin as a control; (**c**) HeLa cells were exposed to 10 MOI of HSV-1/F for 12 h. The cells were then treated with 1 μM of leptomycin B (LMB) or dimethyl sulfoxide (DMSO) for another 2 h. The cells were fixed and stained with antibodies against ICP8 and NOP53, and images were merged. All images were taken using 100× objectives with an Olympus FluoView^TM^. ICP8 localizes in the nucleus and served to demarcate this structure [26]; (**d**) HeLa cells grown in T-25 flasks were transfected with a control (Ctrl) plasmid or plasmids encoding green fluorescent protein (GFP)-tagged wt NOP53 or the nuclear export sequence (NES) deletion mutant (ΔNOP) (5 μg each) for 36 h. The cells were exposed to HSV-1/F at 0.1 MOI for 24 or 48 h. Cell lysates were analyzed by immunoblotting with antibodies against HSV-ICP8, NOP53, and actin; (**e**) HeLa cells were transfected a Ctrl plasmid or plasmids expressing Flag-tagged NOP53 or ΔNOP53. The cells were then mock-infected or infected with HSV-1/F at 1 MOI for 24 h. The cells were harvested, and the nuclear and cytoplasmic fractions were isolated and analyzed by immunoblotting with an antibody against NOP53. Actin and H3 were used as loading controls for cytoplasmic and nuclear proteins, respectively.

It is reported that cytoplasmic translocation of NOP53 has an important function in VSV replication [24]. In accordance with previous reports, it is shown that nucleolar protein NOP53 can be expressed as a set of discrete globular structures within the nuclei of HeLa cells. NOP53 migrated from nuclei to cytoplasm after HSV-1 wild-strain F infection (Figure 1a). To study whether NOP53 translocation is caused by viral replication, cells were infected with an inadequate replication virus—ICP4 deletion mutant virus HSV-1/d120. As shown in Figure 1a, NOP53 did not display obvious translocation in d120-infected cells. To investigate whether viral infection might modulate NOP53 expression in cells, we studied the time-course of NOP53 during HSV-1 infection and found that the NOP53 level was not significantly affected (Figure 1b), indicating that the endogenous level of NOP53 is sufficient to support viral replication. These results suggested that the translocation of NOP53 is related to HSV-1 replication.

To further address the relationship between NOP53 translocation and viral replication, leptomycin B (LMB)—a well-known inhibitor of protein transport [32]—was utilized in this study. LMB can specifically inhibit a broad range of nuclear protein export pathways mediated by CRM1/exportin 1, thus reducing HSV-1 replication [33]. As shown in Figure 1c, NOP53 was entrapped by LMB in the nucleus, suggesting that export of NOP53 from the nucleus involves a CRM1-dependent pathway. A NOP53 mutant that lacks nuclear export sequences (amino acids 358 to 370, and 461 to 472) at the C-terminus of NOP53 (∆NOP53) was then constructed to further verify the relationship between NOP53 translocation and viral replication, as indicated in a previous report [24]. We ectopically expressed green fluorescent protein (GFP)-tagged wild-type (wt) NOP53, GFP-tagged ΔNOP53 or a negative control in cells and then infected them with HSV-1 at an MOI of 0.1. We observed that the levels of viral protein ICP8 (Figure 1d, lanes 4 and 7) were greatly increased by the ectopic expression of wt NOP53. The ectopic expression of ΔNOP53, however, only resulted in subtle increases in ICP8 (lane 6). We concluded that HSV-1 replication priority is a direct consequence of the translocation of NOP53. To verify the cellular localization of the ∆NOP53 mutant upon viral infection, a nuclear and cytoplasmic separation assay was performed. As expected, NOP53 was transported to the cytoplasm of HSV1/F-infected cells, while ∆NOP53 failed to translocate into the cytoplasm (Figure 1e). These results revealed that cytoplasmic translocation of NOP53 plays an important role in HSV-1 replication.

### 3.2. RIG-I-N or Poly (I:C) Stimulation Directly Induces NOP53 Migration

**Figure 2:**
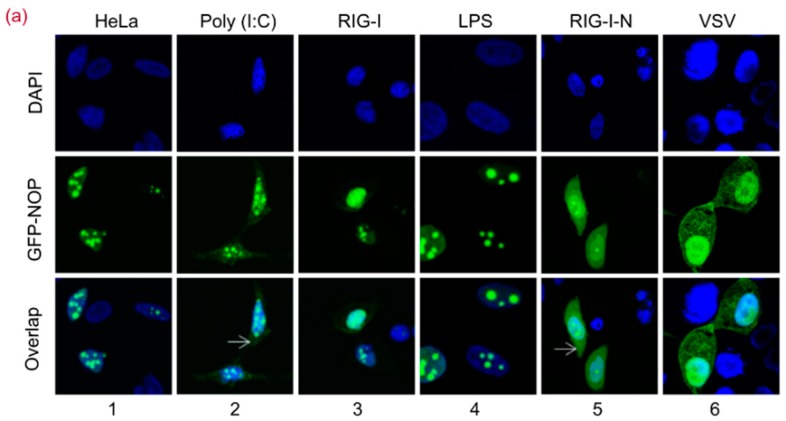

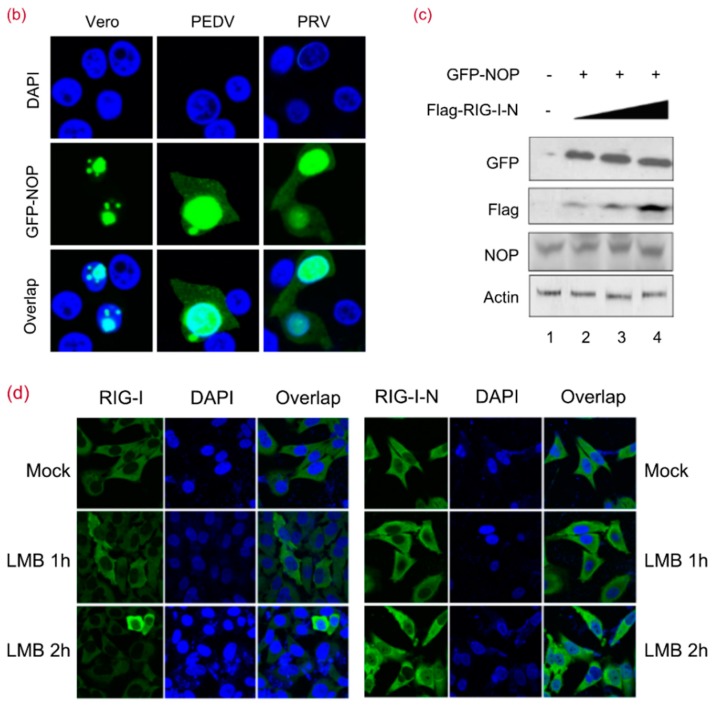
RIG-I-N or Poly (I:C) Stimulation Directly Induces NOP53 Migration. (**a**) HeLa cells in 24-well plate were transfected with plasmids encoding GFP-tagged NOP53, along with poly (I:C), RIG-I, or RIG-I-N (500 ng each) for 24 h or treated with LPS at 100 ng/mL (lanes 1–5). HeLa cells were transfected with plasmids for expressing GFP-tagged NOP53 for 24 h and infected with VSV at an MOI of 5 for 10 h (lane 6). The cells were fixed with 4% paraformaldehyde. Nuclei were stained with DAPI, and NOP was visualized from the fused GFP fluorescence. Images were captured using 100× objectives with an Olympus FluoView™ FV1000; (**b**) Vero cells were transfected with plasmids encoding GFP-tagged NOP53 for 24 h and mock-infected or infected with PEDV or PRV at 10 MOI for 10 h. The cells were fixed with 4% paraformaldehyde. Nuclei were stained with DAPI, and NOP was visualized from the fused GFP fluorescence. Images were captured using 100× objectives with an Olympus FluoView™ FV1000; (**c**) HEK293T cells were co-transfected with GFP-tagged NOP53 expression plasmid and increasing doses of plasmids encoding Flag-tagged RIG-I-N. Protein levels of transfected (GFP) and endogenous (NOP) NOP53 were analyzed by immunoblotting with the indicated antibodies; (**d**) HeLa cells grown in 24-well plates and transfected with plasmids encoding GFP-tagged RIG-I or RIG-I-N (500 ng each) were treated with 1 μM LMB for 1 or 2 h. The cells were fixed with 4% paraformaldehyde. Nuclei were stained with 4′,6-diamidino-2-phenylindole (DAPI), and RIG-I or RIG-I-N was visualized from the fused GFP fluorescence. Images were captured using 40× objectives with an Olympus FluoView™ FV1000.

The dsRNA in cytoplasm is sensed by RIG-I to induce type I IFN activation [6]. We sought to ascertain whether dsRNA affects the translocation of NOP53. As shown in Figure 2a, green fluorescence signals were observed in the cytoplasm and nucleus of the cells. In GFP-tagged NOP53 transfected cells, the signals were mainly distributed in the nuclei (column 1). Consistent with our assumption, the signals were detected in both the cytoplasm and nuclei of cells treated with poly (I:C) (column 2), but this was not RIG-I (column 3) or lipopolysaccharide (LPS) (column 4). Poly (I:C) treatment induced a detectable redistribution of GFP-NOP53 into the cytoplasm, though the majority of GFP-NOP53 was detected in the nucleus.

Given our observation that NOP53 blocked RIG-I-N-induced IFN-β activation [24], we determined whether the translocalization of NOP53 is induced by RIG-I stimulation. Surprisingly, our results showed that the ectopic expression of the constitutively active form of RIG-I (RIG-I-N) induced a redistribution of NOP53 (lane 5). NOP53 is involved in regulating the RLR signaling pathway during viral infection, providing insight into the mechanism of RLR-mediated antiviral responses. After VSV infection (lane 6), fluorescent signals were found in both the cytoplasm and the nuclei, in line with a previous report [24]. We then explored whether PEDV or PRV leads to NOP53 migration as the infection progresses. As shown in Figure 2b, GFP-tagged NOP53 was detectable in the nucleus in Vero cells and then was also detected in the cytoplasm after PEDV or PRV infection. These results supported the conclusion that NOP53 redistribution is a consequence of cellular response to viral replication. Accordingly, targeting the cytoplasmic translocation of NOP53 should be considered in the development of antiviral agents.

To determine whether the ectopic expression of RIG-I-N affects accumulation of NOP53, cells were transfected with increasing amounts of Flag-tagged RIG-I-N and GFP-tagged NOP53. As shown in Figure 2c, the expression level of endogenous and exogenous NOP53 were unaffected. Our results indicated that either RIG-I-N or poly (I:C) stimulation directly induced NOP53 migration without affecting its accumulation. In addition, green fluorescence signals were distributed exclusively in the cytoplasm of GFP-tagged RIG-I or RIG-I-N transfected cells (Figure 2d), regardless of LMB treatment. This result excluded the possibility that RIG-I migrates into nuclei to bind to NOP53.

### 3.3. NOP53 Residues 330–432 Are Necessary for Viral Replication Priority

**Figure 3:**
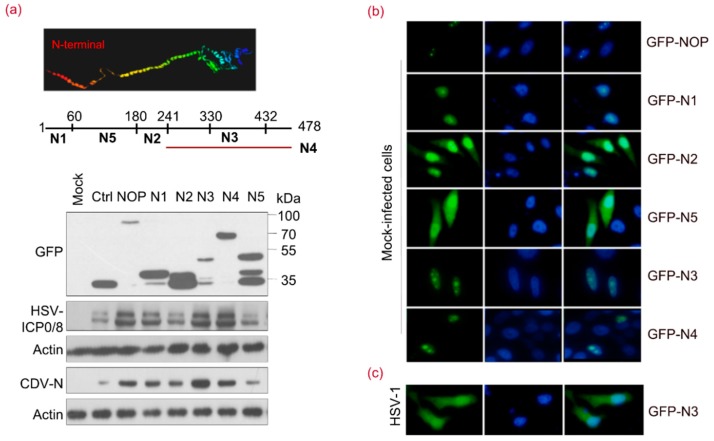
Sequences 330–432 of NOP53 Are Necessary for Viral Replication Priority. (**a**) HeLa cells in T-25 flasks were transfected with a control plasmid (Ctrl) or plasmids encoding GFP-tagged wt NOP53 or truncated NOP53 variants N1, N2, N3, N4, or N5 (5 µg each) for 36 h, followed by infection with HSV-1 or canine distemper virus (CDV) at 0.1 MOI for 48 h. Cell lysates were analyzed by immunoblotting with specific antibodies to detect GFP, HSV-ICP0, HSV-ICP8 and CDV-N, using actin as a control; (**b**) HeLa cells in 24-well plates were transfected with plasmids encoding GFP-tagged wt NOP53 or truncated NOP53 variants N1, N2, N3, N4, or N5 (500 ng each) for 36 h. The cells were fixed and examined with an Olympus microscope; (**c**) HeLa cells transfected with plasmids encoding GFP-tagged N3 were exposed to HSV-1 at 10 MOI for 12 h. The cells were fixed with 4% paraformaldehyde. Nuclei were stained with DAPI. N3 was visualized from the fused GFP fluorescence. Images were captured using 40× objectives with an Olympus IX73 microscope.

To address which putative domains of NOP53 are important for viral replication, we constructed plasmid expression vectors to carry gene fragments encoding GFP-tagged N1, N2, N3, N4, and N5. We transfected cells with these expression vectors individually and consequently exposed the cells to HSV-1 or canine distemper virus (CDV). We detected the ectopic expression of the full length, wt NOP53 enhanced viral replication, as assessed by viral proteins HSV-ICP0/ICP8 and CDV-N. Ectopic expression of N3 (residues 330–432) or N4 (residues 241–478) appeared to enhance viral replication to a level comparable to that by wt NOP53 (Figure 3a). These results indicated that the integrity of the C-terminal region N3 is important for NOP53 to support viral replication.

As GFP-tagged NOP53 and truncated N3 were located in the nucleolus of HeLa cells (Figure 3b), we then evaluated the subcellular localization of N3 in response to HSV-1 infection. As shown in Figure 3c, green fluorescence signals were observed in the cytoplasm and nucleus of N3-transfected cells. These results indicated that N3 variant versus wt NOP53 supported efficient replication of HSV-1 and CDV.

### 3.4. The NOP53-Derived Protein N3-T Can Penetrate into Cells

**Figure 4:**
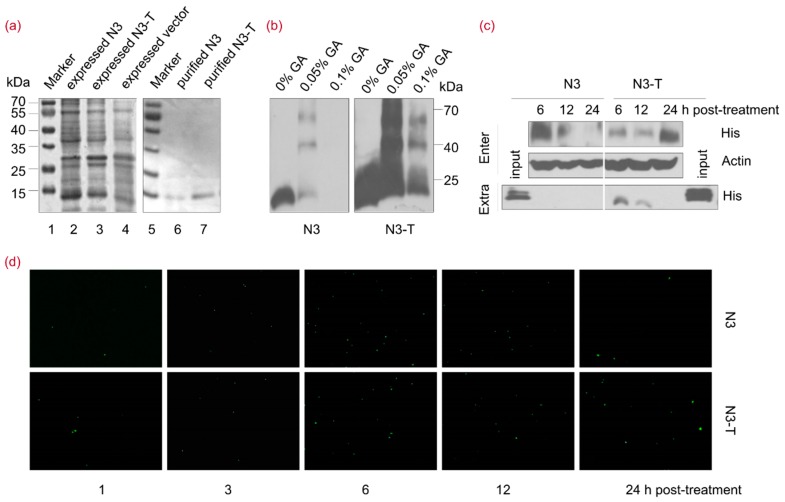
NOP53-derived Protein N3-T Can Penetrate into Cells. (**a**) Lanes 1–7, His-fusion N3 and N3-T proteins were purified using a His-Ni column and the fusion proteins were analyzed by 12% SDS-PAGE. Lane 1, expressed vector control protein; lane 2, expressed His-fusion N3; lane 3, expressed His-fusion N3-T; lanes 4 and 5, protein molecular-weight markers in kDa as indicated; lane 6, purified His-fusion N3; lane 7, purified His-fusion N3-T; (**b**) Purified His-fusion N3 and N3-T proteins were treated with 0%, 0.05%, 0.1% GA for 15 min at 4 °C, followed by SDS-PAGE and immunoblotting with an antibody against His; (**c**) HeLa cells in T-25 flasks were treated with N3 (20 µg/mL) or N3-T (20 µg/mL) for the indicated times. The cells and culture supernatant were harvested for detection using an anti-His antibody; (**d**) HeLa cells in 24-well plate were treated with N3 (20 µg/mL) or N3-T (20 µg/mL) for the indicated times. The cells were fixed and reacted with antibodies against His. Nuclei were stained with DAPI. Images were captured using 10× objectives with an Olympus IX73 microscope.

To further assess the functions of N3 during viral replication, an N3-His fusion protein was expressed in *E. coli* (Figure 4a, lane 2), purified using a Ni-column and eluted with 10% imidazole (lane 6). An N3-T fusion protein containing the cell-penetrating peptide Tat (GGSRYGRKKRRQRRR) [34,35] at the C-terminus, facilitating its ability to penetrate into cells, was also expressed (lane 3) and purified (lane 7) as described above. N3 and N3-T were both highly soluble in phosphate-buffered saline (PBS), with over 85% purity at concentrations of approximately 0.5 and 1 mg/mL, respectively. Chemical cross-linking analysis revealed the self-association of N3 into a trimer structure in the presence of 0.05% glutaraldehyde (GA), whereas N3-T formed trimers when treated with 0.05% or 0.1% GA (Figure 4b).

To estimate the ability of N3 and N3-T to penetrate into cells, cells were treated with either protein at a concentration of 20 µg/mL for 6, 12, or 24 h. Supernatants and cell lysates were prepared separately and analyzed by immunoblotting using an antibody specific to His. As shown in Figure 4c, N3 and N3-T both penetrated into cells. N3 penetrated into cells at an early time point post-treatment and was eliminated after 12 h, whereas N3-T was detectable from 6 to 24 h post-treatment. An indirect immunofluorescence assay was further carried out to verify N3 and N3-T penetrating into cells (Figure 4d). These results indicated that N3-T persists in cells for a longer time than does N3 and that N3-T is more bioavailable than N3.

### 3.5. N3-T Suppresses Type I IFN Antiviral Responses

**Figure 5:**
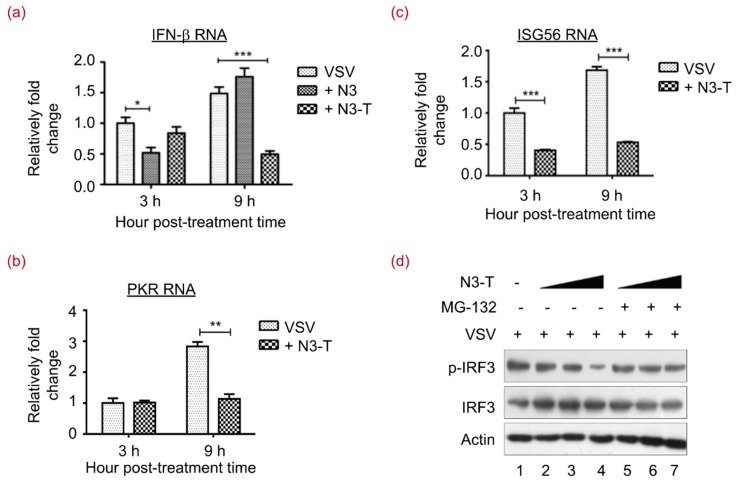
N3-T Suppresses Type I IFN Antiviral Responses. (**a**–**c**) HEK293T cells were infected with VSV at 0.1 MOI for the indicated time in the presence of PBS, N3 (20 µg/mL), or N3-T (20 µg/mL). Total RNAs were isolated and expression levels of IFN-β (**a**); PKR (**b**); and ISG56 (**c**) mRNAs were quantified by real-time polymerase chain reaction (RT-PCR) and normalized to that of glyceraldehyde-3-phosphate dehydrogenase (GAPDH). The bar graphs show the means with standard deviations (SD) of triplicate repeats of one representative experiment. * *p* < 0.05, ** *p* < 0.01, *** *p* < 0.001; (**d**) HEK293T cells were infected with VSV at 0.1 MOI for 24 h in the presence of PBS or increasing doses of N3-T (0, 10, 20 µg/mL). The cells were treated with DMSO (lanes 2–4) or MG-132 (lanes 5–7) for 6 h before collection. Cell lysates were analyzed by immunoblotting with antibodies against p-IRF3 and IRF3. Actin served as the loading control.

Suppression of type I IFN expression by N3-T was verified by examining the mRNA levels of IFN-β, PKR, and ISG56. As shown in Figure 5a, treatment with both N3 and N3-T at 20 µg/mL resulted in reduced IFN-β expression. Our results indicated a 2- or 3-fold decrease in IFN-β mRNA level after 3 h or 9 h of N3 or N3-T, respectively, treatment of cells infected by VSV. Treatment with N3-T protein at 20 µg/mL also resulted in 2–3-fold reductions in PKR (Figure 5b) and ISG56 (Figure 5c) mRNA levels. Phosphorylation of IRF3 on serine 396 has been shown to play a pivotal role in IFN-β induction [11,36]. To investigate the involvement of IRF3 in N3-T suppression of IFN, we treated VSV-infected HEK293T cells with an increasing dose of N3-T in the presence or absence of the proteasome inhibitor MG-132 [37]. Although phosphorylated IRF3 (p-IRF3) was reduced in concert with an increased dose of N3-T (Figure 5d, lanes 2–4), p-IRF3 degradation was blocked by MG-132 (lanes 5–7). This observation suggested that N3-T is able to reduce the p-IRF3 level in cells infected with VSV. All together, these data suggest that suppression of IFN-β is mediated by recombinant protein N3-T to promote viral replication.

### 3.6. N3-T Promotes Viral Replication in a Dose-Dependent Manner

**Figure 6:**
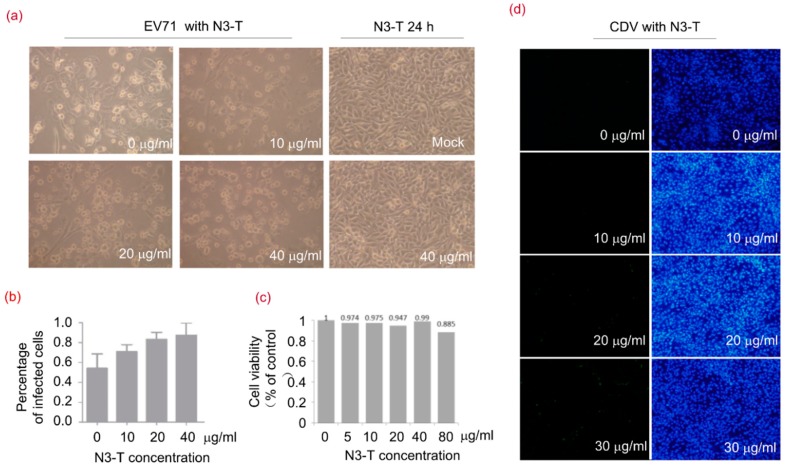
N3-T Promotes Viral Replication in a Dose-Dependent Manner. (**a**,**b**) RD cells were mock-infected or exposed to EV71 of 1 TCID_50_ for 24 h in the presence of PBS or N3-T at the indicated concentrations. The cells were imaged by light microscopy using an Olympus microscope (magnificence 100×) (**a**); and infected cells were quantified from three randomly selected images (**b**); Shown are the means with SD, (**c**) Cells were treated with various concentrations of N3-T for 24 h, and cell viability was determined by the lactate dehydrogenase (LDH) assay; (**d**) Vero cells were exposed to CDV of 0.01 MOI for 36 h in the presence of PBS or N3-T at 0, 10, 20, or 30 µg/mL. The infected cells were imaged by fluorescence microscopy using an Olympus IX73 microscope (magnification of 100×) equipped with a DP73 camera (left) and assessed under light microscopy (panels **c**,**d**). Each image is representative of three experiments (right).

Enterovirus A71 (EV-A71) and CDV were used to investigate the role of N3-T protein in viral replication. As shown in Figure 6a, cells in 6-well plates infected with EV-A71 became shrunken and rounded in appearance and treatment with N3-T increased the number of infected cells. The percentages of infected cells were 54%, 71%, 83%, and 88% when treated with N3-T at concentrations of 0, 10, 20, and 40 µg/mL, respectively (Figure 6b). In addition, cell viability was assessed using a lactate dehydrogenase (LDH) assay, but no significant difference in viability was observed in cells treated with N3-T at concentrations below 40 µg/mL (Figure 6c).

We also quantified the effect of N3-T treatment on CDV infection using an immunofluorescence assay. As shown in Figure 6d, larger numbers of infected cells were observed when treated with N3-T compared to without N3-T treatment. These results demonstrated that recombinant protein N3-T promotes viral replication in a dose-dependent manner.

### 3.7. N3-T Promotes Viral Replication in a Time-Dependent Manner

**Figure 7:**
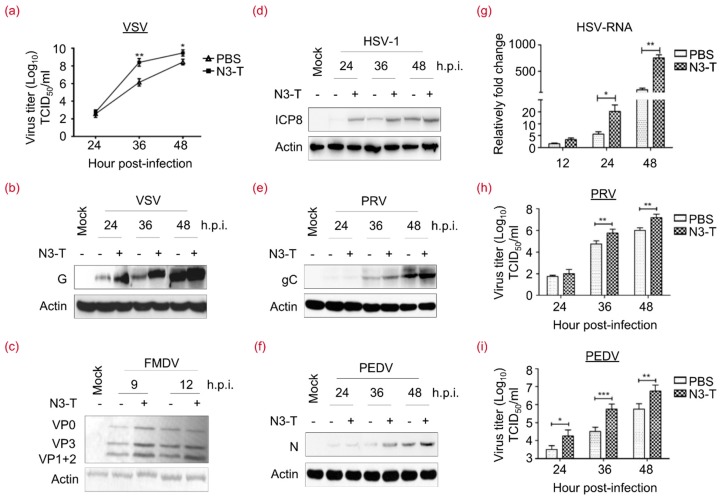
N3-T promotes viral replication in a time-dependent manner. (**a**) HEK293T cells were infected with VSV at 0.1 MOI in the presence of PBS or N3-T (20 µg/mL), and viral yields were determined as TCID_50_/mL at 36, 48, or 60 h. These experiments were performed twice with three replicates in each experiment. Values represent means with SD. * *p* < 0.05; ** *p* < 0.01; (**b**) HEK293T cells in T-25 flask were infected with VSV at 0.01 MOI in the presence of PBS or N3-T (20 µg/mL) for the indicated times. Cell lysates were analyzed by immunoblotting with antibodies against VSV-G. Actin served as the loading control; (**c**) PK-15 cells in T-25 flasks were infected with FMDV at 10 TCID_50_ in the presence of PBS or N3-T (20 µg/mL) for 9 or 12 h. Cell lysates were analyzed by immunoblotting with polyclonal pig antiserum against FMDV and actin. Shown is one representative of two independent experiments; (**d**) HeLa cells were exposed to HSV-1 at 0.01 MOI in the presence of PBS or N3-T (20 µg/mL) for 24, 36, or 48 h. Cell lysates were analyzed by immunoblotting with antibodies against HSV-ICP8 and actin. Shown is one representative of two independent experiments; (**e**,**f**) Vero cells were exposed to PRV (**e**) or PEDV (**f**) at 0.01 MOI in the presence of PBS or N3-T (20 µg/mL) for 24, 36, or 48 h. Cell lysates were analyzed by immunoblotting with antibodies against PRV-gC or PEDV-N, and actin. Shown is one representative of two independent experiments; (**g**) HeLa cells were exposed to HSV-1 at 0.01 MOI in the presence of PBS or N3-T (20 µg/mL) for the indicated times. Total RNAs were isolated and gene expression levels of HSV-ICP8 were quantified by RT-PCR and normalized to that of GAPDH. These experiments were performed twice with three replicates in each experiment. Values represent means of triplicates with SD. * *p* < 0.05, ** *p* < 0.01; (**h**,**i**) Vero cells were exposed to PRV (**h**) or PEDV (**i**) at 10 TCID_50_ in the presence of PBS or N3-T (20 µg/mL) for 24, 36, or 48 h. Virus titer was presented as log_10_ TCID_50_/mL. These experiments were performed twice with three replicates in each experiment. Values represent means of triplicates with SD. * *p* < 0.05, ** *p* < 0.01, *** *p* < 0.001.

To further address the effect of N3-T on viral replication, RNA viruses VSV, porcine epidemic diarrhea virus (PEDV), foot-and-mouth disease virus (FMDV) and DNA viruses HSV-1 and pseudorabies virus (PRV) were utilized. As shown in Figure 7a, we found that N3-T increases viral yield, as assessed with the TCID_50_, by 180- and 10-fold compared with control VSV-infected cells at 36 and 48 h.p.i., respectively. By examining the level of viral proteins VSV-G (Figure 7b), FMDV-VPs (Figure 7c), HSV-ICP8 (Figure 7d), PRV-gC (Figure 7e), and PEDV-N (Figure 7f), we found that N3-T promotes the efficient replication of VSV, FMDV, HSV-1, PRV, and PEDV. To further examine HSV-1 replication following N3-T treatment, RT-PCR was used to elucidate the levels of HSV-ICP8 mRNA. As shown in Figure 7g, there is a 3–5-fold increase in mRNA level. Moreover, virus yields were determined to further examine PEDV and PRV replication. N3-T treatment resulted in an increase of viral yields in TCID_50_ by 17- and 9-fold compared with control PRV-infected cells at 36 and 48 h.p.i., respectively (Figure 7h). While N3-T increases virus yields in TCID_50_ by 9- and 14-fold at 36 and 48 h.p.i., respectively, in comparison with control PEDV-infected cells (Figure 7i). Levels of viral proteins and viral yields were comparably induced by N3-T. These results demonstrated that the N3-T protein can promote the efficient replication of at least 5 laboratory-adapted viruses in a time-dependent manner.

## 4. Discussion

Type I IFN plays an important role in host cell defenses against viral infections. Upon viral infection, host PRRs, such as the RIG-I signaling pathway components, are essential for the recognition of viruses and the initiation of host IFN-mediated antiviral responses [2,3,4]. Upon activation, RIG-I associates with MAVS, resulting in its redistribution to form speckle-like aggregates in cells [38,39]. Our previous studies revealed a nucleolar protein, NOP53, that is involved in IFN-β attenuation via its cytoplasmic translocation to deactivate RIG-I and consequently to promote viral replication [24]. In the present study, NOP53-derived N3-T was expressed and purified to further clarify the importance of cytoplasmic NOP53 for viral replication. N3-T added directly to mammalian cell culture suppressed IFN-β and ISGs expression (Figure 5). Our findings expand the understanding of various negative factors of type I IFN and their underlying mechanisms, especially from the perspective of subcellular localization.

Virus translation and RNA replication depend on nucleo-cytoplasmic trafficking of cellular proteins in infected cells. Several lines of evidence have shown that the redistribution of viral and cellular proteins is critical to viruses and thus may provide new targets for vaccine development and antiviral therapies [40,41]. Viral infection, poly (I:C), or LPS challenge can evoke nuclear export of high-mobility group box (HMGB) proteins [42] and ribonucleoprotein PTB-binding 1 (Raver1) [43]. Interestingly, we discovered that NOP53 migration is directly induced by viral infection, poly (I:C), or RIG-I-N but not by LPS or RIG-I; however, ectopic expression of RIG-I-N, which is located in the cytoplasm, did not affect NOP53 accumulation (Figure 2). These results excluded the possibility that activated RIG-I bound to NOP53 to induce its migration into cytoplasm or sequesters ex novo NOP53 in the cytoplasm. Considering that RIG-I acts as a PRR, we hypothesized that NOP53 redistribution appeared to be involved in general host defense to viral infection. Here, we provide evidence for a novel strategy by which activated RIG-I induces cytoplasmic translocation of a nuclear protein, which has not been described to date. However, the ability of these viruses to induce cytoplasmic translocation of a nuclear protein remains to be addressed.

It is notable that viral infection or RIG-I-N challenge did cause accumulation of endogenous and exogenous NOP53 in cytoplasm (Figure 1 and Figure 2). Viruses have evolved to orchestrate a balanced virus-cell relationship to some extent, including manipulation of the nucleo-cytoplasmic tracking system to exploit protein functions normally localized to a different cellular compartment to facilitate efficient replication. Because the protein level of the tumor suppressor NOP53 is also closely related to DNA damage and repair [22]. It is inferred that the essential level of a cytoplasmic protein in the nucleus would be sufficient for support viral replication.

Several studies have shown that viral proteins may counteract the IFN antiviral response to facilitate replication [44,45]. It is thus inferred that if the accumulation of negative regulators of type I IFN is enhanced, those cells are more susceptible to virus infection than are the parent control cells. In the present study, we established a simple approach to achieving strong virus infection by directly adding recombinant protein N3-T to a culture medium of mammalian cells. The N3-T protein can promote the efficient viral replication of at least 7 laboratory-adapted viruses, including those of the *Rhabdoviridae* (VSV), *Paramyxoviridae* (CDV), *Coronaviridae* (PEDV), *Picornaviridae* (EV-A71, FMDV), and *Herpesviridae* (HSV-1, PRV). This work provides a feasible countermeasure for optimizing viral replication of both enveloped and non-enveloped DNA and RNA viruses (Figure 6 and Figure 7). We expect that N3-T can be used to treat clinical samples of viruses that are fastidious and difficult to cultivate, especially novel clinically isolated and re-emerging viruses.

Our study provides a new route for RIG-I-mediated regulation of subcellular translocation of nucleolar proteins and identifies a function for a nucleolar protein in antiviral innate immunity. However, it remains to be clarified whether NOP53 takes part in additional pathways to support viral replication. It has been reported that NOP53 physically interacts with PTEN, which in turn regulates multiple cellular functions including proliferation, growth, differentiation, and apoptosis [20,21]. Our results showed that the effect of NOP53 on HSV-1 replication is independent of PTEN (Figure S1). Autophagy and apoptosis significantly suppress HSV-1 infection in various cell types [26,46], yet no evidence has been presented to support that NOP53 improves viral replication by suppressing programmed cell death (Figure S2). Understanding the impact of NOP53 conformational changes and corresponding functions upon viral infection is a promising direction for future work.

## Supplementary Materials

The following are available online at http://www.mdpi.com/1999-4915/10/4/208/s1, Figure S1: The effect of NOP53 on HSV-1 replication is independent of PTEN, Figure S2: NOP53 did not affect autophagy and apoptosis induced by HSV-1 infection.
