# Supplementary material for: Cytoplasmic Translocation of Nucleolar Protein NOP53 Promotes Viral Replication by Suppressing Host Defense
Source: Viruses. 2018 Apr 20;10(4):208. doi: 10.3390/v10040208 (PMC5923502; doi:10.3390/v10040208)
Supplement: Supplementary file 1 [file viruses-10-00208-s001.pdf]

Supplementary Information

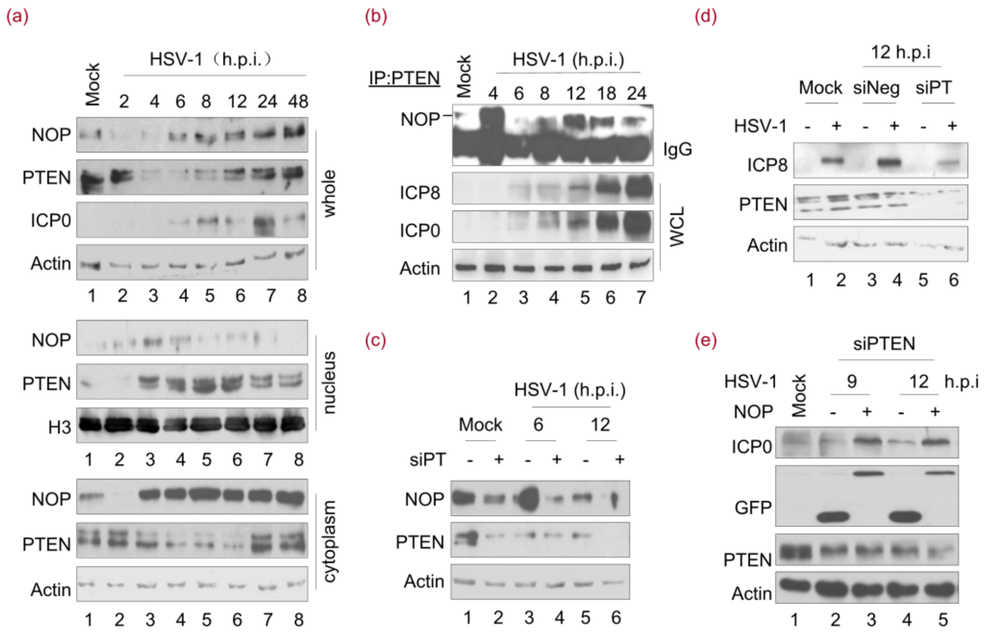

Figure S1. The effect of NOP53 on HSV-1 replication is independent of PTEN. (a) HeLa cells were mock-infected or exposed to HSV-1/F at 10 MOI for the indicated times. Cell lysates were prepared and analyzed by immunoblotting with antibodies directed against NOP53, PTEN, and HSV-ICP0. Actin and H3 served as the loading control. (b) HeLa cells were mock-infected or exposed to HSV-1/F per cell at 10 MOI. Cell lysates were immuno-precipitated with a mouse anti-PTEN antibody and subjected to immunoblotting with specific antibodies to detect NOP. Whole-cell lysates (WCLs) were also examined to confirm the infection of HSV-1/F. (c) HeLa cells were transfected with negative siRNA (siNeg) or specific siRNA targeting PTEN (siPT) for 96 h. The cells were then mock-infected or infected with HSV-1/F at 10 MOI for 12 h, and cell lysates were analysed by immunoblotting using antibodies directed against NOP53, PTEN, and actin. (d) HeLa cells transfected with siNeg or siPT were mock-infected or infected with HSV-1/F at 10 MOI for 12 h. Cell lysates were then analysed by immunoblotting with antibodies against HSV-ICP8 and PTEN. (e) HeLa cells were transfected with siPT followed by transfection of control plasmid or plasmids encoding GFP-tagged NOP53 and infection with HSV-1/F at 10 MOI. Cell lysates prepared 9 or 12 h post infection (h.p.i.) were analysed by immunoblotting using antibodies directed against HSV-ICP0, GFP, PTEN, and actin.

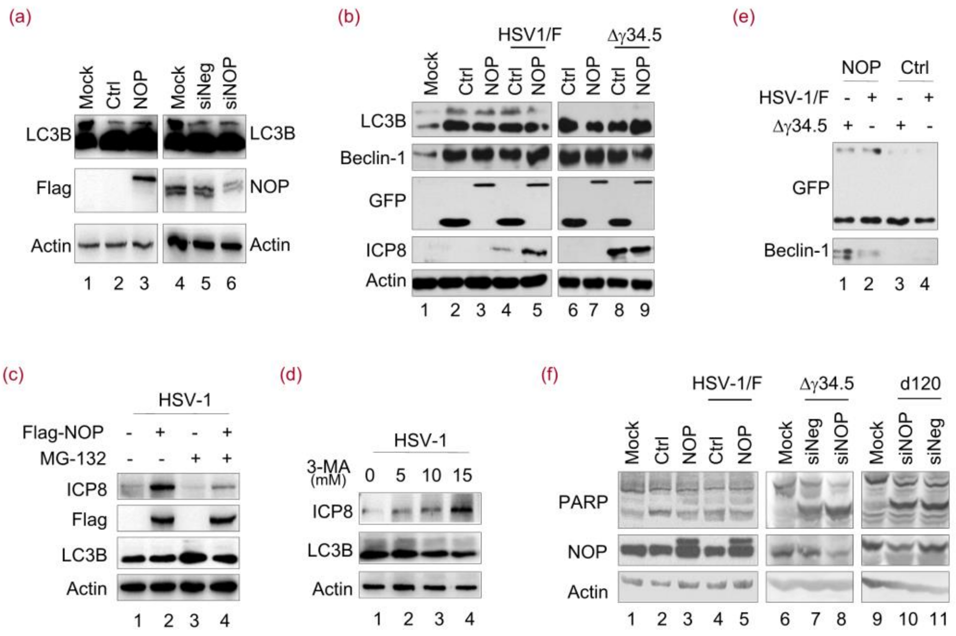

Figure S2. NOP53 did not affect autophagy and apoptosis induced by HSV-1 infection. (a) HeLa cells were either transfected with control plasmid or plasmids encoding Flag-tagged NOP53 or Flag-tagged NOP53-N4 for 36 h (left), or transfected with siNOP or siNeg for 72 h (right). Cell lysates were analyzed by immunoblotting using antibodies directed against LC3B, Flag, and NOP53. Actin served as the loading control. (b) HeLa cells transfected with control or GFP-tagged NOP were mock-infected or either exposed to HSV-1/F or HSV-1/Δγ34.5 at 5 MOI for 20 h. Cell lysates were analyzed by immunoblotting using antibodies directed against Beclin-1, LC3B, GFP, HSV-ICP8 and actin. (c) HeLa cells transfected with control or Flag-tagged N4 were infected with HSV-1/F at 5 MOI for 20 h, in the absence or presence of autophagy inducer MG-132 (1 mM). Cell lysates were analyzed by immunoblotting using antibodies directed against HSV-ICP8, LC3B, Flag, and actin. (d) HeLa cells were infected with HSV-1/F at 5 MOI in the presence of autophagy inhibitor 3-MA at increasing concentrations for 20 h. Cell lysates were analyzed by immunoblotting using antibodies directed against HSV-ICP8, LC3B, and actin. (e) HeLa cells were transfected with control plasmid or plasmids encoding GFP-tagged NOP53. The cells were then infected with HSV-1/F (lanes 2, 4) or HSV-1/Δγ34.5 (lanes 1, 3) at 10 MOI for 20 h. Cell lysates were immuno-precipitated with an anti-GFP antibody and subjected to immunoblotting with specific antibodies to detect GFP and Beclin-1. (f) HeLa cells transfected with control or GFP-tagged NOP53 were mock-infected (lanes 1-3) or infected with HSV-1/F (lanes 4, 5) at 10 MOI. Cells transfected with siNeg or siNOP were mock-infected (lanes 6, 9), or infected with HSV-1/Δγ34.5 (lanes 7, 8) or HSV-1/d120 (lanes 10, 11) at 10 MOI. Cell lysates prepared 14 h.p.i. were then analyzed by immunoblotting using antibodies directed against PARP, NOP53, and actin.
